# Supplementary material for: Detection of Chlorpyrifos Using Bio-Inspired Silver Nanograss
Source: Materials (Basel). 2022 May 11;15(10):3454. doi: 10.3390/ma15103454 (PMC9146306; doi:10.3390/ma15103454)
Supplement: Supplementary file 1 [file materials-15-03454-s001.zip › materials-1687382-supplementary.pdf]

Supplementary Information

Detection of Chlorpyrifos using Bio-Inspired Silver Nanograss

Hyunjun Park [1,†], Joohyung Park [1,†], Gyudo Lee [2,3,*], Woong Kim [4,*] and Jinsung Park [1,*]

[1] Department of Biomechatronics Engineering, Sungkyunkwan University, Suwon 16419, Korea; guswns1105@gmail.com (H.P.); parkjoodori@gmail.com (J.P.)

[2] Department of Biotechnology and Bioinformatics, Korea University, Sejong 30019, South Korea

[3] Interdisciplinary Graduate Program for Artificial Intelligence Smart Convergence Technology, Korea University, Sejong 30019, South Korea

[4] Department of Mechanical Engineering, Hanyang University, Seoul 04763, Korea

* Correspondence: lkd0807@korea.ac.kr (G.L.), oong0331@hanyang.ac.kr (W.K.); nanojspark@skku.edu (J.P.)

† These authors contributed equally to this work.

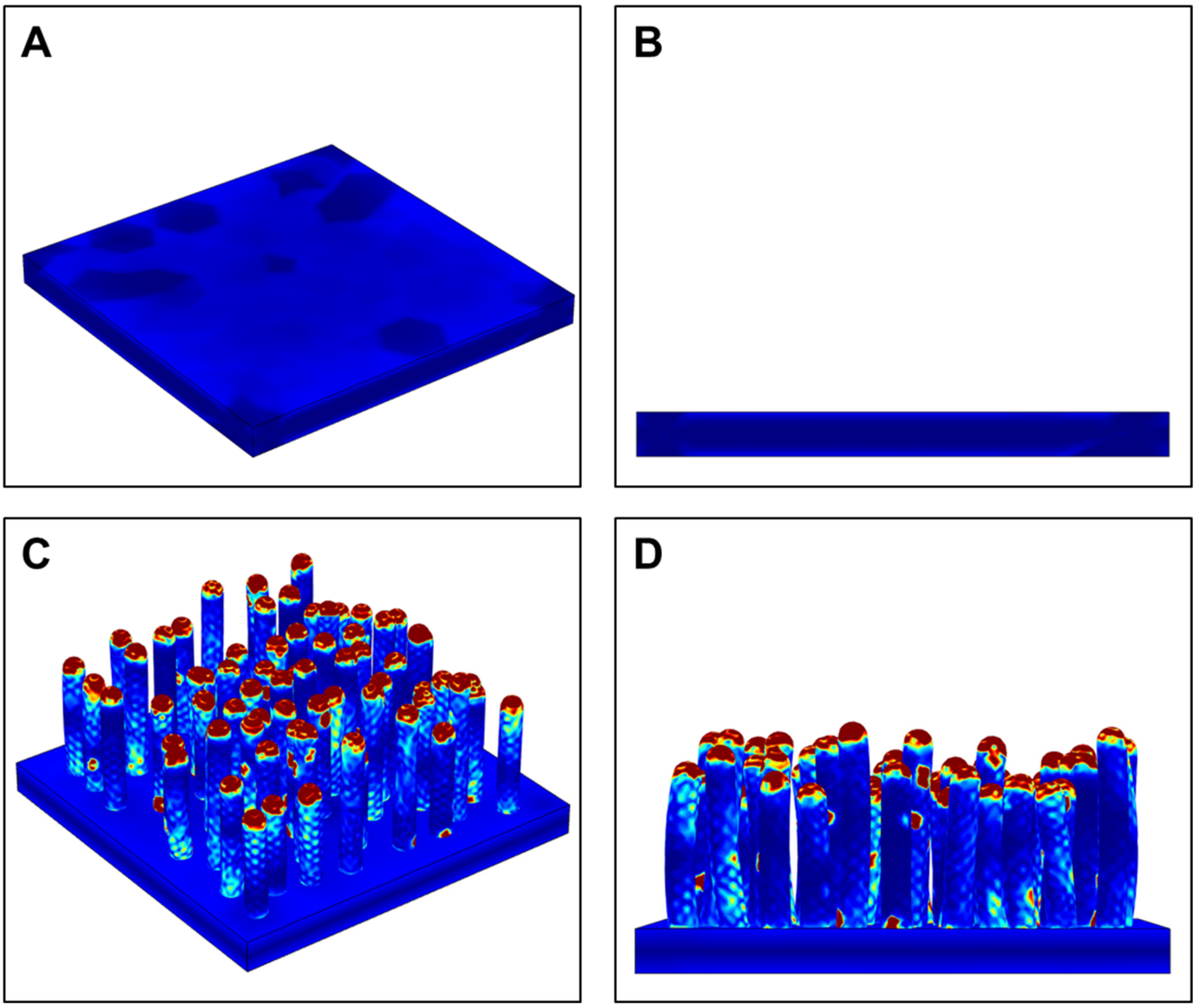

Figure S1. Results of FEM-based electromagnetic simulation for a flat Au surface and Ag-NG. (**A**) Perspective view, (**B**) front view, of the Au surface, (**C**) perspective view, and (**D**) front view, of Ag-NG.

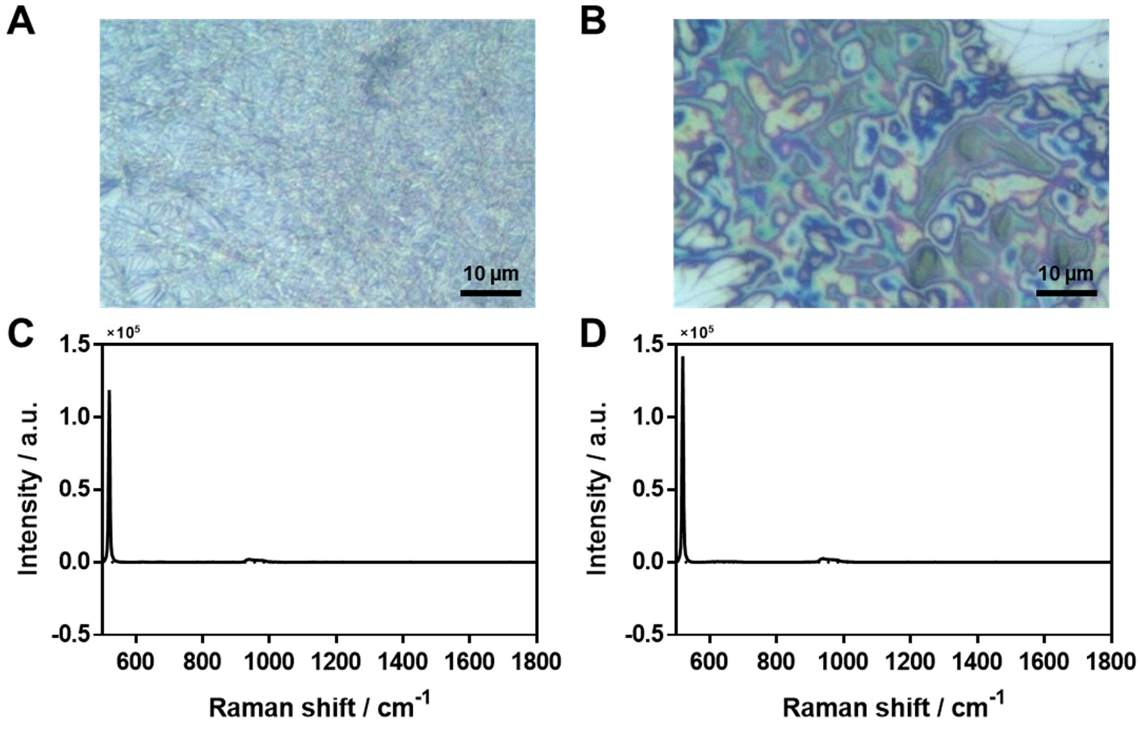

Figure S2. Chlorpyrifos (CPF)-dissolved ethanol sample was studied on a slide glass. Microscopic images of (**A**) control and (**B**) CPF solutions on the slide glass (scale bar: 10 µm). Raman spectra of (**C**) control and (**D**) CPF solutions on a slide glass. None of the samples showed distinguishable spectra.

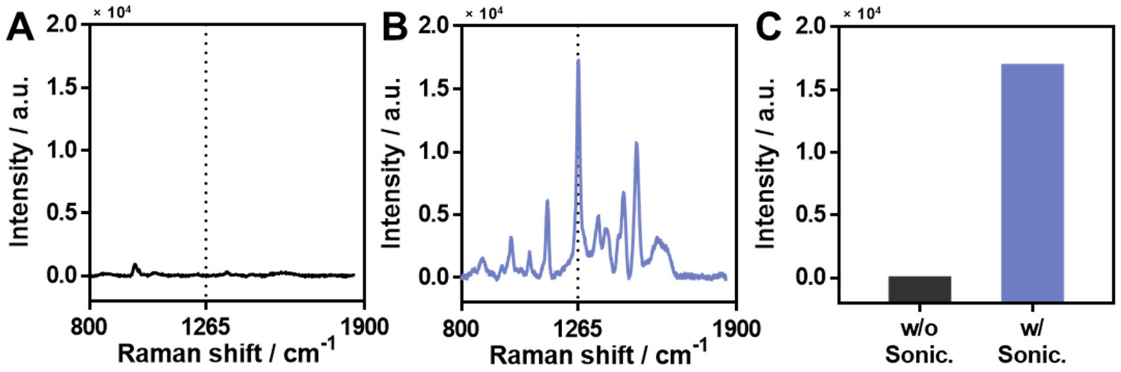

Figure S3. Optimization of sample preparation: Sonication when CPF was dissolved in ethanol. (**A**) Surface-enhanced Raman spectroscopy (SERS) spectrum of un-sonicated CPF solution on Ag-NG. (**B**) SERS spectrum of sonicated CPF solution of Ag-NG. (**C**) Comparison of SERS intensity at 1265 cm^{-1} according to sonication. CPF was dissolved in ethanol and the solution was sonicated for 30 s. The sonicated and unsonicated CPF solutions (1 mM) were treated equally on the Ag-NG.

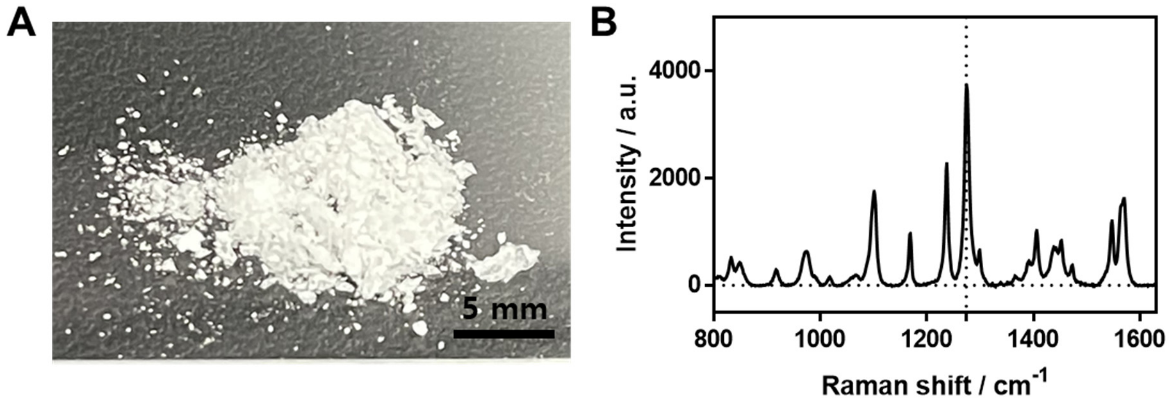

Figure S4. Bulk CPF measurements were used as a control experiment. (**A**) Optical image of CPF powder (scale bar: 5 mm). (**B**) Raman spectrum of CPF powder. The measurement conditions were the same as those in other cases.

Year	Detection methods	LOD	References
2021	LC-MS	2 μM	[1]
2021	Gas sensing array	31.09 nM	[2]
2021	UV-vis	200 nM	[3]
2019	Colorimetric assay	99.83 μM	[4]
2019	Fluorescence	29 nM	[5]
2018	Colorimetric	28.523 nM	[6]
2022	SERS (Ag-NG)	500 nM	This work

Table. S1. Comparison with other sensing techniques such as mass spectroscopy, colorimetry, fluorescence, and UV-vis

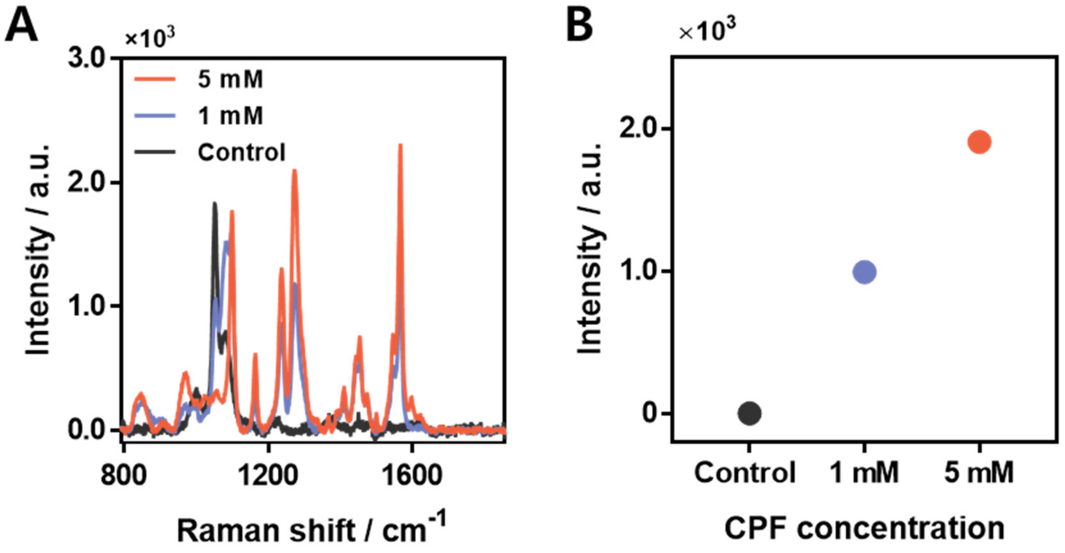

Figure. S5. The CPF detection in the tap water by Ag-NG. (**A**) Spectra of CPF according to the concentrations. (**B**) the SERS intensities of CPF at 1265 cm^{-1}.

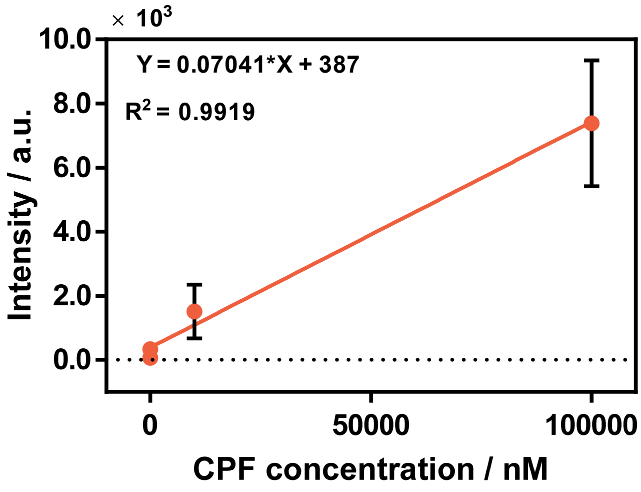

Figure. S6. Linear fitting results about the SERS intensities at 1265 cm-1 according to the CPF concentration. (R2 = 0.9919)
